# Diosmin Attenuates Methotrexate-Induced Hepatic, Renal, and Cardiac Injury: A Biochemical and Histopathological Study in Mice

**DOI:** 10.1155/2017/3281670

**Published:** 2017-07-27

**Authors:** Mohamed M. Abdel-Daim, Hesham A. Khalifa, Abdelrahman Ibrahim Abushouk, Mohamed A. Dkhil, Saleh A. Al-Quraishy

**Affiliations:** ^1^Pharmacology Department, Faculty of Veterinary Medicine, Suez Canal University, Ismailia, Egypt; ^2^Department of Pharmacology, Faculty of Veterinary Medicine, Zagazig University, Zagazig, Egypt; ^3^Faculty of Medicine, Ain Shams University, Cairo, Egypt; ^4^Department of Zoology, College of Science, King Saud University, Riyadh, Saudi Arabia; ^5^Department of Zoology and Entomology, College of Science, Helwan University, Cairo, Egypt

## Abstract

The current study was designed to investigate the beneficial role of diosmin, a biologically active flavonoid, against methotrexate- (MTX-) induced hepatic, renal, and cardiac injuries in mice. Male Swiss albino mice received a single intraperitoneal injection of MTX (at 20 mg/kg, body weight) either alone or in combination with oral diosmin (at 50 or 100 mg/kg body weight, for 10 days). Serum was used to evaluate tissue injury markers, while hepatic, renal, and cardiac tissue samples were obtained for determination of antioxidant activity as well as histopathological examination. Diosmin treatment ameliorated the MTX-induced elevation of serum alkaline phosphatase, aminotransferases, urea, creatinine, lactate dehydrogenase, and creatine kinases as well as plasma proinflammatory cytokines (interleukin-1-beta, interleukin-6, and tumor necrosis factor-alpha). Additionally, both diosmin doses significantly reduced tissue levels of malondialdehyde and nitric oxide and increased those of glutathione, glutathione peroxidase, glutathione reductase, glutathione S-transferase, superoxide dismutase, and catalase, compared to the MTX-intoxicated group. Histopathological examination showed that diosmin significantly minimized the MTX-induced histological alterations and nearly restored the normal architecture of hepatic, renal, and cardiac tissues. Based on these findings, diosmin may be a promising agent for protection against MTX-induced cytotoxicity in patients with cancer and autoimmune diseases.

## 1. Introduction

Methotrexate (MTX) is a widely known folate antimetabolite agent, used to treat several types of cancer [1] and autoimmune diseases [2]. Using MTX at cyclic high doses to treat malignant tumors can be associated with severe hepatotoxicity and acute renal failure [3, 4], while its chronic use at lower doses may cause progressive liver fibrosis, uremia, and hematuria [4, 5]. Moreover, few articles have reported an acute toxic effect of MTX on the cardiovascular system [6]. Methotrexate-induced cytotoxicity can be a product of the interaction of several factors including the patient's risk factors, type of disease, dosing schedule, and treatment duration, as well as the presence of genetic apoptotic factors [7].

Long-term use of MTX leads to accumulation of its intracellular storage form “MTX polyglutamates,” which has been suggested as a mechanism for MTX hepatotoxicity [8]. It also inhibits the cytosolic nicotinamide adenosine diphosphate- [NAD(P)-] dependent dehydrogenases, decreasing the cellular availability of NADP [9], which is normally used by glutathione reductase (GR) to maintain the reduced state of glutathione (GSH) [10]. Moreover, its high affinity to dihydrofolate reductase inhibits the production of thymidylate, suppressing DNA synthesis [11]. Despite these side effects, MTX is a highly effective chemotherapeutic agent; therefore, research efforts should be directed at preventing and treating its cytotoxicity.

**Figure 1:**
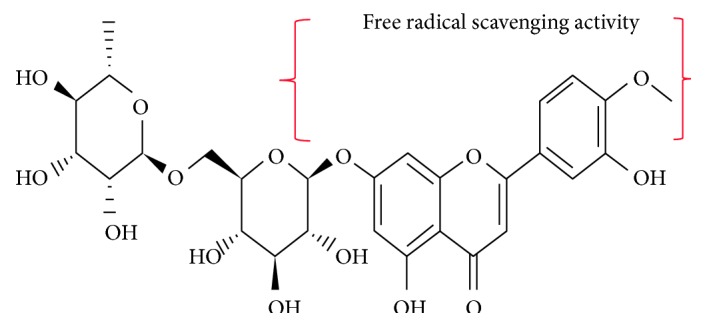
The chemical structure of diosmin. The area (in brackets) refers to the main antioxidant component of diosmin molecule.

Diosmin (3′,5,7-trihydroxy-4′-methoxyflavone 7-rutinoside) is an unsaturated flavonoid glycoside, present in citrus fruits (Figure 1) [12]. It is a biologically active polyphenol that has anti-inflammatory [13], antioxidant, antihyperglycemic [14], and antimutagenic properties [15]. In rat models, Ahmed and colleagues showed a nephroprotective role for diosmin against alloxan-induced nephropathy [16], while Tahir et al. highlighted its beneficial effect against alcoholic liver injury [13]. Moreover, diosmin could successfully improve cardiac function [17] and exert antihyperlipidemic effects against isopropanol-induced myocardial injury in rats [18].

A literature survey retrieved no scientific reports on the protective effects of diosmin against MTX toxicity. Therefore, in light of the above inferences from other evidence, we designed this study to investigate the beneficial role of diosmin against MTX-induced injuries to mouse hepatic, renal, and cardiac tissues.

## 2. Materials and Methods

### 2.1. Chemicals

Methotrexate was purchased from Shanxi PUDE Pharmaceutical Company (Shanxi, China), while diosmin was purchased from Sigma (St. Louis, MO, USA). All the assay kits were purchased from Biodiagnostics Co. (Cairo, Egypt), except for the kits of creatine kinase (CK) and CK-myoglobin binding (CK-MB) (Stanbio™, TX, USA), lactate dehydrogenase (LDH) enzymes (Randox Laboratories Ltd., UK), tumor necrosis factor- (TNF-) *α* (BioSource International Inc., Camarillo, CA, USA), interleukin- (IL-) 1*β*, and IL-6 (Glory Science Co. Ltd., Del Rio, TX, USA).

### 2.2. Mice

Thirty-two adult male Swiss albino mice, weighing 22 to 27 g, were obtained from the Egyptian Organization for Biological Products and Vaccines. The animals were first acclimatized for seven days at the experiment site (animal house of the Department of Pharmacology, Faculty of Veterinary Medicine, Suez Canal University) under optimal environmental conditions (12-hour light-dark cycles, temperature [20 ± 2°C] with moderate humidity [60 ± 5%]). All the animal handling procedures, used in this study, were approved by the Research Ethical Committee at the Faculty of Veterinary Medicine, Suez Canal University, Egypt (Approval number 201608).

### 2.3. Experimental Design

The mice were divided into four groups, each of eight. Mice in group I served as a negative control, receiving saline only during the entire experimental period. Mice in group II received a single intraperitoneal injection of MTX at a dose of 20 mg/kg bw (mimicking the acute exposure to an acute large dose in humans) [19]. Mice in groups III and IV received oral diosmin for 10 days at doses of 50 and 100 mg/kg bw [20], respectively. On the sixth day of the experiment, mice in groups III and IV received i.p MTX injection, at the same dose used in group II mice.

All animals were sacrificed on the 11th day after blood sample collection via direct cardiac puncture. The liver, heart, and kidneys were dissected into two parts: the 1st part was fixed with 10% formalin for further histopathological examination and the 2nd part was used for biochemical analysis. For the latter purpose, tissue pieces (each of 0.5 g) were washed with cold normal saline and homogenized in 2.5 volumes of ice-cold 0.1 M potassium phosphate buffer (pH 7.4). The resulting homogenate underwent two cycles of centrifugation at 600*g* and 10000*g*, and the supernatant was collected and stored at −70°C to measure the tissue content of oxidative biomarkers and antioxidant enzymes.

### 2.4. Serum Biochemical Analysis

The obtained blood samples were allowed to clot for 30 minutes then centrifuged at 3000 rpm for 15 minutes in order to obtain clear sera. The sera were then stored at −20°C for further biochemical analysis. The methods described by Reitman and Frankel [21] to measure the serum levels of alanine transferase (ALT) and aspartate transferase (AST) and those described by Kind and King [22] to measure the serum level of alkaline phosphatase (ALP) were used. To estimate the serum levels of renal injury markers, we used the methods described by Larsen for measurement of serum creatinine [23] and those described by Coulombe and Favreau for measurement of serum urea [24]. In addition, the cardioprotective effect of diosmin was evaluated enzymatically by estimating the serum activity of CK and CK-MB, using Stanbio CK-NAC (UV-Rate)/CK-MB kits (TX, USA), according to the methods described by Szasz et al. [25] and Würzburg et al. [26], respectively. Later, serum levels of LDH were measured according to Babson SR and Babson AL [27].

### 2.5. Evaluation of Tissue Lipid Peroxidation, Nitric Oxide, and Antioxidant Enzymes

For evaluation of lipid peroxidation, the methods described by Uchiyama and Mihara were used to measure the hepatic, cardiac, and renal tissue content of malondialdehyde (MDA) [28]. The concentration of nitric oxide (NO) in these tissues was assessed according to Green et al. [29]. Later, tissue levels of reduced GSH, superoxide dismutase (SOD), and catalase (CAT) were determined according to the methods described by Beutler et al. [30], Nishikimi et al. [31], and Aebi [32], respectively. Moreover, we measured tissue levels of glutathione S-transferase (GST), GR, and glutathione peroxidase (GPx) according to Habig et al. [33], Zanetti [34], and Paglia and Valentine [35], respectively.

### 2.6. Estimation of Proinflammatory Cytokines

Enzyme-linked immunosorbent assay (ELISA) kits were used to measure the serum levels of TNF-*α* (BioSource International Inc., Camarillo, CA, USA), IL-1*β*, and IL-6 (Glory Science Co. Ltd., Del Rio, TX, USA). The measurements were performed according to the manufacturer's instructions, and absorbance was read using an automated ELISA reader at 420 nm.

### 2.7. Histopathological Examination

Using a rotatory microtome, 5 *μ*m thick sections were sliced from the liver, heart, and kidneys for histopathological examination. These sections were later stained with hematoxylin-eosin (H&E) dye (Merck) and examined at 200x magnification using a power light microscope (Zeiss, Germany). We used a semiquantitative analysis to assess the tissue injury index in examined sections. The results were expressed as the sum of individual score grades (0: no findings, 1: mild, 2: moderate, or 3: severe) for each of the following parameters: degeneration, cellular swelling, cellular vacuolization, necrosis, congestion, and hemorrhage.

### 2.8. Statistical Analysis

We used SPSS (Statistical Package for Social Sciences) software (version 20) to perform the statistical analysis. All values were expressed as the mean and the standard error of mean (SEM). The means of different groups were compared using the one-way analysis of variance (ANOVA), followed by Tukey's post hoc comparison tests. A *p* value <0.05 was considered statistically significant.

## 3. Results

### 3.1. Biochemical Findings

#### 3.1.1. Serum Concentrations

Intraperitoneal injection of MTX significantly increased serum concentrations of AST, ALT, and ALP enzymes, compared to normal control levels (*p* < 0.01). However, treatment of MTX-injected mice with diosmin (at 50 and 100 mg/kg bw) significantly reduced the elevation of AST levels by 37.3% and 55.6%, ALT levels by 41.3% and 58.1%, and ALP levels by 36.2% and 46.4%, respectively. Treatment of MTX-injected mice with 100 mg/kg of diosmin restored serum concentrations of these parameters to normal control levels.

Similarly, MTX-injected mice showed a significant increase in serum concentrations of cardiac (CK and CK-MB enzymes) as well as renal injury markers (urea and creatinine) in comparison to normal control mice (*p* < 0.01). Upon treatment of MTX-injected mice with diosmin (at 50 and 100 mg/kg bw), we recorded significant reductions in elevated serum levels of LDH by 21.2% and 30.2%, CK by 44% and 63%, CK-MB by 53% and 76%, urea by 47.1% and 64.1%, and creatinine by 42.2% and 73%, respectively. As shown before, only the 100 mg/kg dose of diosmin could restore serum concentrations of these parameters to normal control levels (Table 1).

**Figure 2:**
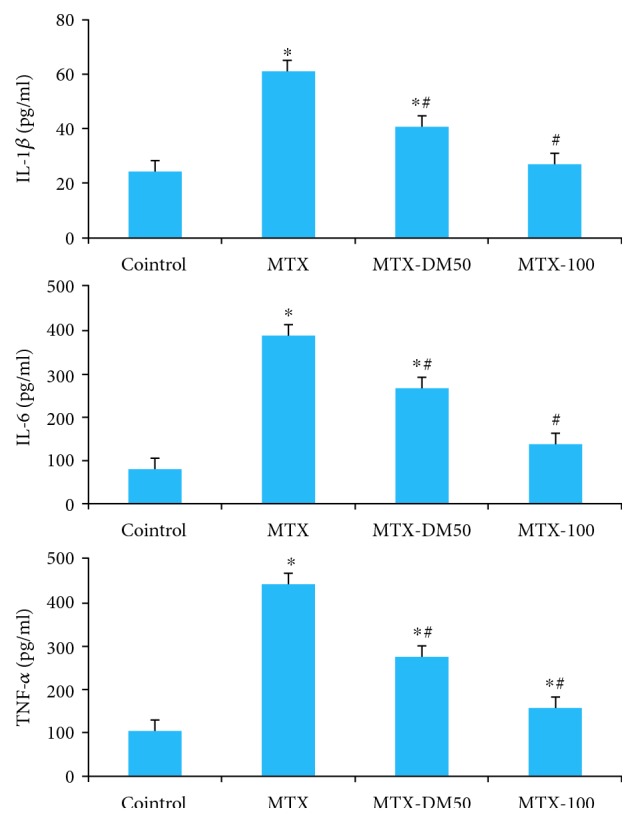
Effect of MTX with or without diosmin on proinflammatory cytokines IL-1*β*, IL-6, and TNF-*α*. DM: diosmin; IL: interleukin; MTX: methotrexate; TNF: tumor necrosis factor. ^∗^Significant change at *p* < 0.01 with respect to the negative control group. ^#^Significant change at *p* < 0.01 with respect to the MTX group as the positive control group.

Plasma concentrations of inflammatory markers (IL-1*β*, IL-6, and TNF-*α*) were significantly higher (*p* < 0.01) in the MTX group than in the normal control group. Consistent with previous findings, diosmin treatment (at 50 and 100 mg/kg bw) significantly reduced plasma concentrations of IL-1*β* by 33.6% and 56.1%, IL-6 by 31.5% and 65.1%, and TNF-*α* by 38% and 64.2%, respectively. Interestingly, restoration of IL-1*β* and IL-6 plasma concentrations in MTX-injected mice to normal control levels was noted only in the 100 mg/kg diosmin group; however, neither doses (50 and 100 mg/kg bw) could restore the plasma concentration of TNF-*α* in MTX-injected mice to normal levels (Figure 2()).

#### 3.1.2. Antioxidant Activity in Isolated Tissue Samples

Biochemical analysis of hepatic, cardiac, and renal tissues in MTX-injected mice showed a marked reduction in GSH concentration and antioxidant enzymes' levels (GST, GR, GPx, SOD, and CAT), as well as significant increases in MDA and NO tissue concentrations (*p* < 0.01). Administration of diosmin significantly ameliorated the elevations of MDA and NO tissue concentrations in MTX-injected mice (*p* < 0.01). Moreover, it could increase tissue concentrations (*p* < 0.05) of GSH, as well as the activities of GST, GR, GPx, SOD, and CAT enzymes (Table 2).

### 3.2. Histopathological Findings

#### 3.2.1. Liver

**Figure 3:**
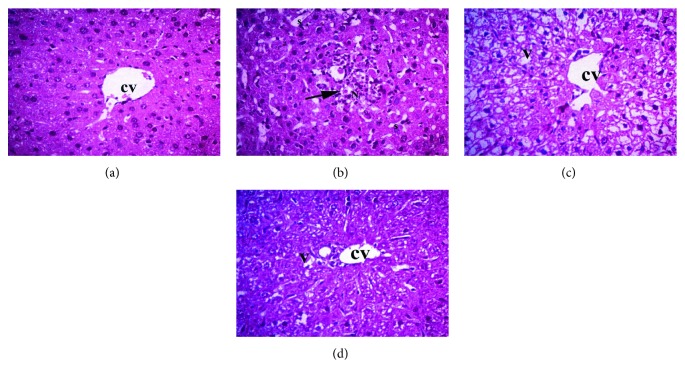
Histopathologic examination of liver tissue by H&E at ×400. CV: central vein; V: vacuolar degeneration; S: sinusoids. The arrow indicates necrosis and lymphocytic infiltrations. Groups are (a) normal liver tissue in the negative control group, (b) MTX group, (c) MTX + diosmin 50 mg/kg group, and (d) MTX + diosmin 100 mg/kg group.

Liver tissue samples from the negative control group showed normal hepatic cells, arranged in cords around normal central veins. However, liver tissue samples from the MTX group showed focal areas of necrosis around the central vein, sinusoidal dilatation, vacuolar degeneration, and focal infiltration with leukocytes, mainly lymphocytes. In group III (MTX + diosmin 50 mg/kg), moderate hepatocyte vacuolization, sinusoidal dilatation, and partial disruption of hepatic cords' arrangement were observed, while group IV (MTX + diosmin 100 mg/kg) liver sections showed remarkable improvements of all MTX-induced histological changes (Figure 3()).

#### 3.2.2. Heart

**Figure 4:**
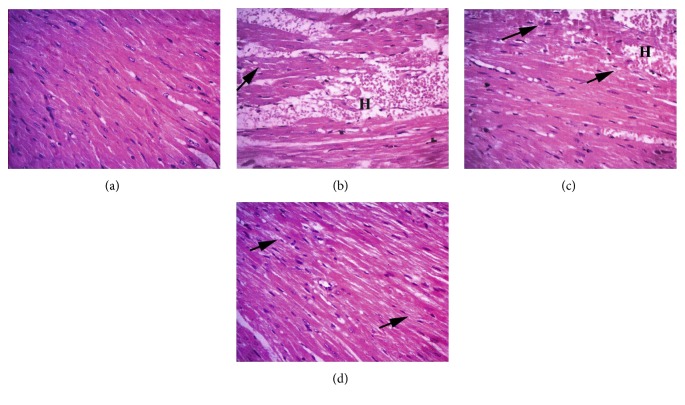
Histopathologic examination findings of cardiac tissue sections by H&E at ×400. Arrows point to myocardiocyte degeneration. H: hemorrhage. Groups are (a) control group showing normal myocardium, (b) MTX group, (c) MTX + diosmin 50 mg/kg group, and (d) MTX + diosmin 100 mg/kg group.

Cardiac tissue microscopic slices from the negative control group showed normal histological appearance of cardiomyocytes with normal cigar-shaped nuclei. Conversely, we observed massive degenerative changes, cardiomyocyte necrosis, severe congestion, intermuscular edema, and intermuscular hemorrhage in mice that received MTX alone. In group III (MTX + diosmin 50 mg/kg), we detected mild-to-moderate intermuscular hemorrhage and edema, while group IV (MTX + diosmin 100 mg/kg) cardiac muscles showed minimal intermuscular edema with restoration of the normal cardiac tissue architecture (Figure 4).

#### 3.2.3. Kidney

**Figure 5:**
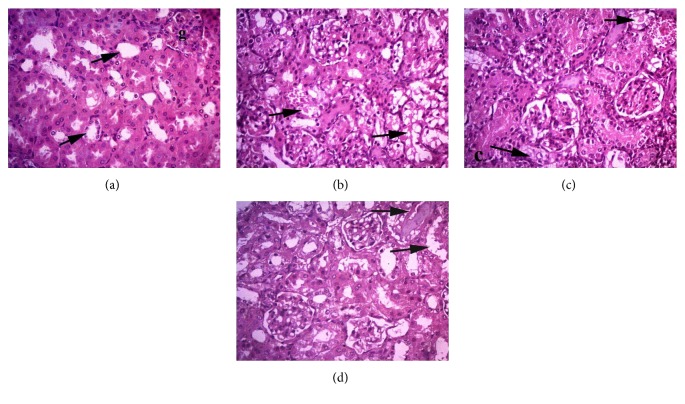
Histopathologic examination findings of kidney tissue by H&E at ×400. Arrows point to renal tubules. Groups are (a) control group showing normal renal tubules, (b) MTX group showing severe vacuolation, (c) MTX + diosmin 50 mg/kg group showing moderate degeneration, and (d) MTX + diosmin 100 mg/kg showing mild degeneration.

Microscopic examination of H&E-stained renal tissue slices from the negative control group showed normal histological appearance with tubular arrangement of epithelial cells. On the contrary, tissue samples from the kidneys of group II mice (MTX) showed intensive degenerative changes and swelling of epithelial cells of the proximal tubules. Minimal areas of necrosis with pyknotic and karryorrhetic nuclei, as well as focal areas of severe vacuolar degeneration of proximal convoluted tubules, were also observed. In group III (MTX + diosmin 50 mg/kg), we observed a moderate-to-severe swelling of tubular epithelial cells along with areas of mild vacuolar degeneration, while group IV (MTX + diosmin 100 mg/kg) microscopic renal slices showed an improvement in tubular structure and minimal cellular swelling, compared to those of the MTX group (Figure 5()).

Quantitative analysis showed that in comparison to the control group, MTX administration caused significantly higher tissue lesion scores (*p* < 0.01) in all examined organs. Interestingly, treatment with diosmin (both doses) caused a significant decrease in all tissue lesion scores, when compared to the MTX group (Table 3).

## 4. Discussion

This study highlights—for the first time—the protective role of diosmin against MTX-induced injuries to mouse hepatic, renal, and cardiac tissues. In experimental mice, diosmin was able to significantly reduce the biochemical and histological alterations, induced by MTX. These findings are mostly mediated via its antioxidant, anti-inflammatory, and antiapoptotic effects as shown by the decrease in oxidative and nitrative stress markers (MDA and NO), enhanced antioxidant defense mechanisms (GSH, SOD, and CAT), reduced serum levels of inflammatory cytokines (IL-1*β*, IL-6, and TNF-*α*), and the reduction in necrosis and vacuolar degeneration on histopathological examination after diosmin treatment.

The cytotoxic effects of MTX on the liver and kidneys were investigated in previous reports. Chladek et al. showed that MTX is metabolized in the liver by an enzymatic system [36]. The increased level of its cellular metabolites could induce oxidative and inflammatory tissue damages [37], which could be demonstrated in the form of histopathological changes [38] and elevation of serum liver enzymes [39]. Moreover, MTX metabolites were found to precipitate in renal tubules, causing marked nephrotoxicity in rats [40, 41].

Methotrexate can induce oxidative stress through inhibition of NADP synthesis, which is used by GR to maintain the reduced state of GSH. It can also induce nitrative stress by increasing the levels of TNF-*α* and IL-1*β*, which increase the expression of inducible nitric oxide synthase (iNOS) and the production of NO and cyclic guanosine monophosphate (cGMP) [42]. Moreover, TNF-*α* can directly induce cytotoxicity to hepatocytes and glomerular and epithelial cells of the proximal convoluted tubules [43, 44]. In addition, IL-1*β* stimulates the synthesis of prostaglandin E2 in renal mesangial cells, changing the glomerular hemodynamics [45], while IL-6 increases the expression of fibronectin, causing thickening of the glomerular basement membrane [46].

Our study showed that diosmin mitigated MTX-induced oxidative and nitrative stresses by increasing GSH levels, lowering NO levels, and enhancing the activity of antioxidant enzymes. Tahir et al. showed that diosmin could alleviate ethanol-induced liver injury by reducing the activation of nuclear factor kappa B (NF-*κ*B) and inhibiting the expression of TNF-*α*, iNOS, and cyclooxygenase-II [13]. Rehman et al. used kidney injury molecule-1 (KIM-1), a highly sensitive biomarker for renal injury, to evaluate the protective role of diosmin against trichloroethylene (TCE) nephrotoxicity [47]. In agreement with other studies [48, 49], they reported that TCE administration increased the levels of renal KIM-1, while adding diosmin to TCE could effectively reduce these elevations.

To our knowledge, there is no study about the histological effects of MTX on the cardiac tissue. However, our data showed that MTX induces marked cardiac tissue damage in mice as indicated by the previously mentioned histopathological changes and elevation of plasma levels of cardiac enzymes (CK and CK-MB). These biochemical and histological changes were significantly reduced after diosmin treatment. In the same vein, previous reports showed the ability of diosmin to protect against isopropanol-induced myocardial infarction (at a daily dose of 5 to 10 mg/kg bw) and ischemia-/reperfusion-related cardiac dysfunction (at a daily dose of 50 to 100 mg/kg bw), as evidenced by restoration of normal echocardiographic patterns and reduction in plasma levels of cardiac troponins [17, 18]. Diosmin can offer additional cardioprotection through its antihyperlipidemic (via inhibition of hepatic HMG CoA reductase), antihyperglycemic [14], and antihypertensive effects [50].

Histopathological analysis showed that MTX administration induced an acute inflammatory reaction, marked by vascular congestion, tissue edema, and infiltration by leukocytes. This reaction was explained in earlier studies by the ability of MTX to induce oxidative and nitrative stresses, as well as activation of nuclear factor-*κ*B (NF-*κ*B) and p38 pathways [19, 51]. Several degenerative changes were also noted, such as pyknotic and karryorrhetic nuclei, cellular vacuolization (reflecting dilatation of the smooth endoplasmic reticulum), and disturbance of tissue architecture.

The observed reduction in cellular necrosis and vacuolar degeneration following diosmin treatment may be related to (a) reduction in DNA damage indirectly through alleviation of MTX-induced oxidative stress and inflammation and (b) the possible antiapoptotic role of diosmin, demonstrated in previous experimental studies. For example, Liu et al. reported that diosmin protected against cerebral ischemia/reperfusion injury in mice through activation of the JAK2/STAT3 signaling pathway, a key regulator of cellular proliferation [52]. Moreover, Rehman et al. showed that diosmin offered nephroprotection in TCE-intoxicated mice by reducing the expression of Bax, p53, caspase-3, caspase-7, and caspase-9 [47].

Interestingly, several animal studies showed an antimutagenic effect of diosmin against esophageal [53], hepatic [54], colon [55], and buccal pouch tumors [56]. Rajasekar et al. concluded that diosmin (at a daily dose of 100 mg/kg bw) suppressed cellular proliferation and tumor progression in 7,12-dimethylbenz(a)anthracene- (DMBA-) induced hamster buccal pouch carcinoma through inhibition of the IL-6/STAT3 signaling pathway [56]. Additionally, Tahir et al. and Tanaka et al. attributed the antimutagenic effect of diosmin to inhibition of cellular proliferation and downregulation of inflammatory markers [54, 55]. Therefore, adding diosmin to MTX does not only protect against its cytotoxicity but may also provide synergistic antimutagenic effects.

Based on our results, as well as those of previous reports, diosmin can exert its antioxidant effect directly (by free radical scavenging [57]) and indirectly (by upregulating cellular antioxidant enzymes, such as GR, GPx, CAT, and SOD). Earlier investigations have shown that several antioxidants, such as N-acetylcysteine [58, 59], carvacrol [60, 61], curcumin [38, 62], and silymarin [63], can protect against MTX-induced hepatic and renal injury in animal models. Future studies should directly compare diosmin to these antioxidants to determine the optimal chemoprotective agent in this regard and whether diosmin has additional cytoprotective effects against MTX.

An earlier report by Iqbal and colleagues showed that MTX-induced nephrotoxicity can be ameliorated by folinic acid administration [64]; therefore, the effects of adding folinic acid as a supplement to diosmin should be investigated. Future studies should also test the protective role of diosmin against other chemotherapeutic agents. The antimutagenic and the cytoprotective effects of diosmin against chemotherapeutic drugs have been studied separately; therefore, further research should investigate the effects of diosmin on animal models with malignant tumors, receiving chemotherapeutic agents.

## 5. Conclusion

In experimental mice, diosmin significantly reduced the biochemical as well as the histological alterations, induced by MTX due to its antioxidant and anti-inflammatory activities. Therefore, diosmin may be a promising agent for protection against MTX-induced cytotoxicity in patients with cancer and autoimmune diseases. Future studies should investigate the effects of diosmin on animal models with malignant tumors and its potential for cytoprotection against other chemotherapeutic agents.

## Figures and Tables

**Table 1:** Ameliorative effects of diosmin on serum concentrations of hepatic, cardiac, and renal injury markers in mice exposed to methotrexate.

Group	AST	ALT	ALP	LDH	CK	CK-MB	Urea	Creatinine
	(U/ml)	(U/ml)	(U/l)	(U/l)	(U/l)	(U/l)	(mg/dl)	(mg/dl)
Control	30.7 ± 1.8	27.2 ± 1.3	68.3 ± 1.6	290.5 ± 7.3	116 ± 3.1	36.6 ± 1.3	22.3 ± 1.5	0.41 ± 0.02
MTX	83 ± 4.6^∗^	71.5 ± 2.7^∗^	132.7 ± 3.03^∗^	440.7 ± 10.3^∗^	394 ± 17.6^∗^	190.3 ± 15.6^∗^	73.5 ± 3.5^∗^	2.1 ± 0.15^∗^
MTX-DM50	52 ± 4.7^∗^^#^	42 ± 2^∗^^#^	84.6 ± 2.5^∗^^#^	347.2 ± 15.7^∗^^#^	221 ± 11.6^∗^^#^	89.4 ± 4.3^∗^^#^	39 ± 2.3^∗^^#^	1.2 ± 0.13^∗^^#^
MTX-DM100	36.9 ± 2.8^#^	30 ± 1.4^#^	71.2 ± 2.6^#^	307.6 ± 9^#^	146 ± 9.7^#^	45.7 ± 4.3^#^	26.4 ± 2.4^#^	0.57 ± 0.04^#^

Values are means ± SEM. ^∗^Significant change at *p* < 0.01 with respect to the negative control group. ^#^Significant change at *p* < 0.01 with respect to the MTX group as the positive control group. ALP: alkaline phosphatase; ALT: alanine transferase; AST: aspartate transferase; CK: creatine kinase; LDH: lactate dehydrogenase.

**Table 2:** Ameliorative effects of diosmin on tissue concentrations of lipid peroxidation and antioxidant markers in mice exposed to methotrexate.

Group	MDA (nmol/g)	NO (*μ*mol/g)	GSH (mg/g)	GST (U/g)	GR (U/g)	GPx (U/g)	SOD (U/g)	CAT (U/g)
*I. Hepatic tissue concentrations*								
Control	28.9 ± 1.8	53.3 ± 2.9	0.41 ± 0.02	49.13 ± 1.62	43.95 ± 1.45	37.78 ± 1.64	116.4 ± 3.99	41.39 ± 3.16
MTX	84.1 ± 3.07^∗^	104.8 ± 2.9^∗^	0.16 ± 0.02^∗^	19.84 ± 1.74^∗^	17.98 ± 0.93^∗^	15.92 ± 1.75^∗^	42.2 ± 1.28^∗^	19.9 ± 2.24^∗^
MTX-DM50	66.2 ± 4.5^∗^^#^	83.3 ± 2.5^∗^^#^	0.23 ± 0.02^∗^	32.26 ± 2.08^∗^^#^	28.32 ± 1.99^∗^^#^	24.38 ± 1.94^∗^^#^	84.8 ± 3.59^∗^^#^	30.07 ± 2.52
MTX-DM100	35.2 ± 2.5^#^	58.2 ± 3.6^#^	0.33 ± 0.02^#^	43.01 ± 1.28^#^	37.45 ± 1.31^∗^^#^	28.70 ± 3.92^∗^^#^	106.04 ± 4.25^#^	39.33 ± 2.37^#^

*II. Cardiac tissue concentrations*								
Control	49.47 ± 2.7	57.1 ± 2.2	26.8 ± 1.0	33.58 ± 1.78	5.76 ± 0.34	16.69 ± 0.96	57.32 ± 2.3	3.1 ± 0.14
MTX	138.7 ± 5.2^∗^	158.7 ± 5.5^∗^	12.35 ± 0.6^∗^	16.65 ± 0.57^∗^	2.45 ± 0.13^∗^	7.20 ± 0.29^∗^	29.58 ± 1.1^∗^	0.73 ± 0.05^∗^
MTX-DM50	89.6 ± 3.4^∗^^#^	97.5 ± 3.4^∗^^#^	16.44 ± 1.2^∗^	24.42 ± 0.92^∗^^#^	3.67 ± 0.18^∗^^#^	10.62 ± 0.62^∗^^#^	44.29 ± 2.4^∗^^#^	1.3 ± 0.08^∗^^#^
MTX-DM100	56.3 ± 3.4^#^	62.2 ± 3.4^#^	24.3 ± 1.2^#^	32.19 ± 1.13^#^	5.59 ± 0.28^#^	15.48 ± 0.18^#^	53 ± 2.4^#^	2.7 ± 0.2^#^

*III. Renal tissue concentrations*								
Control	36.8 ± 2.42	43.8 ± 1.47	9.04 ± 0.12	9.39 ± 0.34	13.06 ± 0.48	6.16 ± 0.14	57.2 ± 1.24	4.3 ± 0.27
MTX	77.3 ± 5.02^∗^	87.6 ± 3.6^∗^	4.52 ± 0.30^∗^	5.23 ± 0.27^∗^	6.51 ± 0.40^∗^	2.96 ± 0.19^∗^	27.55 ± 1.9^∗^	2.05 ± 0.31^∗^
MTX-DM50	49.5 ± 3.13^#^	63.6 ± 2.45^∗^^#^	6.04 ± 0.43^∗^^#^	7.24 ± 0.20^∗^^#^	9.30 ± 0.28^∗^^#^	4.76 ± 0.16^∗^^#^	43.9 ± 3.9^∗^^#^	3.46 ± 0.27^#^
MTX-DM100	39.2 ± 2.2^#^	45.9 ± 1.04^#^	8.25 ± 0.18^#^	9.26 ± 0.26^#^	12.08 ± 0.33^#^	6.02 ± 0.15^#^	53.5 ± 2.6^#^	4.09 ± 0.28^#^

Values are means ± SEM. ^∗^Significant change at *p* < 0.01 with respect to the negative control group. ^#^Significant change at *p* < 0.01 with respect to the MTX group as the positive control group. CAT: catalase; MDA: malondialdehyde; NO: nitric oxide; GPx: glutathione peroxidase; GR: glutathione reductase; GSH: glutathione; GST: glutathione S-transferase; SOD: superoxide dismutase.

**Table 3:** Organ lesion scores in control and different experimental groups.

Lesion	Experimental groups			
	Control	MTX	MTX-DM50	MTX-DM100
Hepatic lesions	0.00	3.00 ± 0.00^∗^	1.80 ± 0.13^∗^^#^	1.20 ± 0.13^∗^^#^
Renal lesions	0.00	3.00 ± 0.00^∗^	2.30 ± 0.15^∗^^#^	1.90 ± 0.31^∗^^#^
Cardiac lesions	0.00	3.00 ± 0.00^∗^	2.00 ± 0.21^∗^^#^	1.40 ± 0.16^∗^^#^

Data are expressed as means ± SEM. ^∗^Significant change at *p* < 0.01 with respect to the negative control group. ^#^Significant change at *p* < 0.01 with respect to the MTX group as the positive control group.

## Abbreviations

ALP: Alkaline phosphatase
ALT: Alanine transferase
AST: Aspartate transferase
CK: Creatine kinase
CK-MB: Creatine kinase-myocardial B fraction
IL: Interleukin
LDH: Lactate dehydrogenase
MTX: Methotrexate
TNF: Tumor necrosis factor.
